# Celecoxib-Loaded Solid Lipid Nanoparticles for Colon Delivery: Formulation Optimization and In Vitro Assessment of Anti-Cancer Activity

**DOI:** 10.3390/pharmaceutics14010131

**Published:** 2022-01-05

**Authors:** Hamdan N. Alajami, Ehab A. Fouad, Abdelkader E. Ashour, Ashok Kumar, Alaa Eldeen B. Yassin

**Affiliations:** 1 Pharmaceutical Services Administration, King Saud Medical City, Ministry of Health, Riyadh 12746, Saudi Arabia; 2 Department of Pharmaceutics, Faculty of Pharmacy, Assiut University, Assiut 71526, Egypt; ehab.ahmed1@pharm.aun.edu.eg; 3 Department of Basic Medical Sciences, Kulliyyah of Medicine, International Islamic University Malaysia, Kuantan 25200, Pahang Darul Makmur, Malaysia; aeashour@iium.edu.my; 4 Vitiligo Research Chair, College of Medicine, King Saud University, Riyadh 12372, Saudi Arabia; asukhmant@ksu.edu.sa; 5 Pharmaceutical Sciences Department, College of Pharmacy, King Saud bin Abdulaziz University for Health Sciences, Riyadh 14611, Saudi Arabia; 6 King Abdullah International Medical Research Center, National Guard Health Affairs, Riyadh 11481, Saudi Arabia

**Keywords:** solid lipid nanoparticles, ultrasonic melt-emulsification, celecoxib, HT29, MTT, zeta-potential

## Abstract

This work aimed to optimize a celecoxib (CXB)-loaded solid lipid nanoparticles (SLN) colon delivery system for the enhancement of anticancer activity. An ultrasonic melt-emulsification method was employed in this work for the preparation of SLN. The physical attributes were characterized for their particle sizes, charges, morphology, and entrapment efficiency (%EE), in addition to DSC and FTIR. The in vitro drug release profiles were evaluated, and the anticancer activity was examined utilizing an MTT assay in three cancer cell lines: the colon cancer HT29, medulloblastoma Daoy, and hepatocellular carcinoma HepG2 cells. All of the prepared SLN formulations had nanoscale particle sizes ranging from 238 nm to 757 nm. High zeta-potential values (mv) within −30 s mv were reported. The %EE was in the range 86.76–96.6%. The amorphous nature of the SLN-entrapped CXB was confirmed from SLN DSC thermograms. The in vitro release profile revealed a slow constant rate of release with no burst release, which is unusual for SLN. Both the F9 and F14 demonstrated almost complete CXB release within 24 h, with only 25% completed within the first 5 h. F9 caused a significant percentage of cell death in the three cancer cell lines tested after 24 h of incubation and maintained this effect for 72 h. The prepared CXB-loaded SLN exhibited unique properties such as slow release with no burst and a high %EE. The anticancer activity of one formulation was extremely significant in all tested cancer cell lines at all incubation times, which is very promising.

## 1. Introduction

Celecoxib (CXB) is a selective inhibitor of the COX-2 enzyme and it is and has been approved by the Food and Drug Administration (FDA) for the treatment of the symptoms of osteoarthritis and rheumatoid arthritis [1,2,3]. CXB has also been shown to have significant chemo-preventive activity in colon carcinogenesis and breast cancer [1,2,3]. COX-2 expression in cancer cells has been demonstrated in a number of animal models [4]. A number of studies have shown that CXB, either alone or in combination with cetuximab, increases tumor cell apoptosis of human colorectal cancer in a mouse xenograft model [5,6,7]. CXB has recently been approved by the FDA to treat Familial Adenomatous Polyposis (FAP) based on a clinical study that showed a reduction of 28% in colorectal polyps [8]. According to the biopharmaceutical classification system, it is a class II drug. Many attempts have been made to overcome CXB’s poor solubility and increase its dissolution rate from various dosage forms, thereby increasing overall bioavailability [9,10,11].

Loading CXB into nano-particulate systems such as polymeric, liposomal, nano-crystals and polymer/inorganic hybrid nanoparticles has been extensively used in the literature to improve drug efficacy for a variety of therapeutic applications such as anterior and posterior eye disorders, ulcerative colitis, melanoma and brain tumor [12,13,14,15,16,17,18]. Ibrahim et al. [13] demonstrated that ophthalmic in situ gels containing CXB-loaded chitosan, alginate or polycaprolactone nanoparticles successfully maintained high levels of CXB in all eye tissues for an extended period of time while preventing drug systemic absorption. Margulis et al. [12] used a murine model of myocardium infarction to report a remarkable improvement in the vascularization of ischemic myocardium induced by CXB-polymeric nanoparticles. They proposed that CXB could have a new therapeutic indication in angiogenesis. CXB and plumbagin co-encapsulated nano-liposomes were tested for anticancer activity against xenograft melanoma tumor by Gowda et al. [16]. The combination inhibited tumor growth by up to 72 percent without causing any detectable toxicity. Wu et al. [15] used biotin and heparin with calcium carbonate and phosphate salts to create polymer/inorganic hybrid nanoparticles co-encapsulated with CXB and buthionine sulfoximine. Through downregulation of both GSH and P-gp, this combination demonstrated high efficacy in reversing multiple drug resistance (MDR) in the resistance cells MCF-7/ADR [15]. Gugulothu et al. [18] demonstrated the efficacy of co-encapsulated curcumin/CXB nanoparticles in the treatment of ulcerative colitis (UC) in a UC-rat model. CXB has been shown in numerous studies to be beneficial in a variety of cancer tumors [19,20,21,22].

SLN are matrix nano-particulate systems composed primarily of lipids with melting points higher than ambient temperature that allow them to remain solid. The type of lipids, their content and their percentage all play an important role in determining the efficacy of the SLN formulation [23]. Triglycerides (e.g., tricaprin), partial glycerides (e.g., Imwitor), fatty acids (e.g., stearic acid), steroids (e.g., cholesterol) and waxes are examples of commonly used lipids (e.g., cetylpalmitate). Emulsifiers are used to prevent particle agglomeration in lipid dispersion. They are essential in the release of drugs from the SLN matrix. The ability of SLN to be produced on an industrial scale using high-pressure homogenization and spray drying techniques, which have been used in the pharmaceutical industry [24,25], is a significant advantage.

SLN have proven to be an appealing alternative carrier system to traditional colloidal systems such as liposomes, micro-emulsions and polymeric nanoparticles [23,24,25]. They combine some of the benefits of polymeric nanoparticles and liposomes. Unlike polymeric nanoparticles, they do not cause local tissue irritation and chronic toxicity. They also could overcome the stability problems of liposomes and could enhance the residence time of drugs in the gastrointestinal tract, which results in enhanced bioavailability [25,26,27,28]. Furthermore, SLN can enhance the bioavailability of some drugs by the inhibition of an efflux mechanism inside the gastrointestinal tract [29]. The SLN provide a great promise as drug carriers of anticancer drugs by increasing anticancer activity with an increasing accumulation of drugs inside tumor cells [30]. The aim of this work is to optimize CXB loading into an orally administered SLN system capable of localizing CXB in the colon and utilizing nanoparticulate beneficial properties in enhancing anticancer activity.

## 2. Materials and Methods

Celecoxib (CXB) was purchased from FDC Limited, Maharashtra, India. Softisan 154, Dynasan 114, Imwitor 308 were purchased from Sasol Germany GmbH (Witten, Germany). Tween 80, Sodium deoxycholate, stearic acid and MTT (3-(4,5-dimethyl-thiazol-2-yl)-2,5-diphenyltetrazolium bromide) were obtained from Sigma–Aldrich Chemical Company (St. Louis, MO, USA). DMEM/high glucose, DMEM/F12, fetal bovine serum (FBS), penicillin/streptomycin, L-glutamine, non-essential amino acids and HEPES reagent were purchased from Gibco, Invitrogen (Eugene, OR, USA). Additionally, 96-well plates, T-25 and T-75 flasks, as well as serological pipettes and pipette tips were obtained from TPP Techno Plastic Products AG (Trasadingen, Switzerland). Cremophor EL was purchased from BASF, Ludwigshafen, Germany. All other chemicals were of analytical grade.

### 2.1. Preparation of Solid Lipid Nanoparticles

A simple ultrasonic melt-emulsification method was used for SLN preparation. Briefly, 50 mg CXB was dissolved in a certain amount of melted lipids at a temperature 10 °C above its melting point in a water bath. Simultaneously, 5 mL of aqueous surfactant/co-surfactant solution, heated in the same water bath, was mixed with CXB lipid solution using probe sonicator (Bandelin Sonopuls HD220, Bandelin Electronics, Berlin, Germany) for 3 min at 40% voltage efficiency. The formed emulsion was then dispersed in 20 mL of chilled water using a magnetic stirrer for 3 min. The formed SLN were collected by centrifugation at 50,000 RCF for 30 min at 4 °C and washed by re-centrifugation for another cycle in chilled water. The residue was dispersed in 2 mL 5% dextrose and stabilized by lyophilization using a freeze drier machine (Alpha 1–4 LD-2, Martin Christ, Osterode, Germany) over a period of 72 h at −59 °C and 0.090 mbar [31]. Table 1 shows the composition of each SLN formulation.

### 2.2. Analytical Method

A simple-sensitive HPLC method with UV detection was applied with minor modifications [32]. The HPLC system consisted of a Waters Model 1515 HPLC pump and a Waters dual absorbance UV detector (Waters Inc., Bedford, MA, USA). A C18 analytical μ-Bondapack column (150 mm length × 4.6 mm i.d., 10 μm particle size) was utilized for the separation of CXB using an isocratic elution of a mobile phase consisting of acetonitrile: water (60:40) at 1 mL/min flow rate. The detector was adjusted at λ = 260 nm.

### 2.3. Evaluation of the Prepared SLN

#### 2.3.1. Particle Size and Polydispersity Evaluation

The mean particle size and polydispersity of the SLN were measured by means of photon correlation spectroscopy using a 90 Plus particle-size analyzer, Brookhaven Instruments Corporation, (Holtsville, NY, USA). The SLN formulations were diluted at a ratio of 1: 1000 *v/v* with distilled water before testing. The angle of detection was 90° and the temperature was 25 °C. The obtained values represented the mean of three measurements with a standard error.

#### 2.3.2. Measurement of Zeta Potential

The SLN surface charge was evaluated for each sample using the same above Brookhaven instrument by applying the Laser Doppler Velocimetry (LDV) mode.

#### 2.3.3. Measurement of Drug Entrapment and Drug Loading 

The entrapment efficiency (%EE) and drug loading (%DL) of CXB in SLN formulations were determined by centrifugation of the different samples at 50,000 RCF at 4 °C for 30 min. The non-entrapped CXB in the supernatant was determined by HPLC. Then, %EE and %DL of CXB were calculated according to the following formulae:

(1) $$\%\mathrm{EE}=\frac{W\;\mathrm{entrapped}\;\mathrm{drug}\;}{W\;\mathrm{initial}\;\mathrm{drug}}\;\times \;100$$

(2) $$\%\mathrm{DL}=\frac{W\;\mathrm{entrapped}\;\mathrm{drug}\;}{\left(W\;\mathrm{total}\;\mathrm{drug}\;+\;W\;\mathrm{of}\;\mathrm{total}\;\mathrm{lipid}\;+\;W\;\mathrm{total}\;\mathrm{surfactants}\right)}\times 100$$

where W represents the weight in mg.

#### 2.3.4. Differential Scanning Calorimetry

The Shimadzu DSC-60 (Shimadzu Corporation, Kyoto, Japan) was employed to detect the thermal behavior of CXB in pure and SLN entrapped states. The samples were weighed (between 5 and 8 mg) and sealed in aluminum pans. After calibration with Indium/zinc standard, each pan was heated at a constant rate of 10 °C/min over a temperature range of 25–210 °C. An inert atmosphere was maintained through a nitrogen gas flow of 50 mL/min.

#### 2.3.5. Fourier Transform Infrared Spectroscopy

Fourier transform infrared (FTIR) spectrum was studied on a Perkin Elmer FTIR instrument (Perkin Elmer, Waltham, MA, USA) using the potassium bromide (KBr) disc technique. The samples were scanned against a blank KBr pellet background, where the baseline was corrected. Samples were scanned at a wave number ranging from 4000 to 400 cm^−1^ with a resolution of 1.0 cm^−1^ [33,34].

#### 2.3.6. Particle Morphology

A scanning electron microscope (JSM-6360 LV, JEOL, Tokyo, Japan) was used for studying the surface morphology of the prepared SLN. Samples of SLN formulation were mounted on a carbon tape using a gold palladium layer in a high-vacuum evaporator. Then, the samples were scanned, and photos were taken at an acceleration voltage of 20 kV.

### 2.4. In Vitro Release Profile Study

A certain weight from each formula equivalent to 1 mg CXB was dispersed in 1 mL of phosphate buffer (pH = 6.8) and was inserted into a cellulose dialysis tube with a typical molecular weight cut-off of 12–14 KD, sealed from one end. After sealing the other end, the tube was immersed in a beaker containing 40 mL of phosphate buffer pH 6.8. The beakers were incubated in a shaker water bath adjusted at 37 °C ± 0.5 and a speed of 80 RPM. Aliquots of 10 mL were removed at certain time intervals and were replaced by an equal volume of fresh buffer in order to maintain a sink condition. Finally, the drug released was determined using the HPLC.

### 2.5. Release Kinetic Analysis

The fraction CXB released (Mt/M∞) was fitted with time t according to the Higushi diffusion model as well as the Korsemeyer–Peppas model using the following equations:

Higuchi diffusion equation

Mt/M∞ = k_h_ t^½^ (3)

where k_h_, is the Higuchi diffusion rate constant

Peppas and Korsemayer equation

Mt/M∞ = kp t^n^ (4)

where kp is the release rate constant at the elapsed time t. The exponent n is a constant, indicating the mechanism of release; where n ≤ 0.45 indicates Fickian diffusion, 0.45 ≤ n ≤ 0.89 indicates non-Fickian (anomalous) diffusion, n = 0.89 indicates case II transport, and n ≥ 0.89 indicates Super Case II transport. The data fitting was done by the MULTI computer program. Then, the date was examined according to the sum of the squared residuals (SSR) and a comparison of the Akaike’s information criterion (AIC) [35]. The AIC and SSR were calculated using Equations (5) and (6):

AIC = n [ln (SSR)] + 2p(5)

SSR *=* ΣΣ*Wij*(*Ci,j − ∫* (tj, p))2 (6)

where n, is the number of experimental points, and p is the number of parameters to be estimated.

### 2.6. Cell Lines

The hepatocellular carcinoma HepG2, the human colorectal cancer HT-29, and medulloblastoma Daoy cells were purchased from The American Type Culture Collection (ATCC, Manassas, VA, USA). HepG2 and HT-29 cells were cultured in DMEM/high glucose with 10% heat-inactivated fetal bovine serum (FBS), 2 mM L-glutamine, and 1% penicillin–streptomycin. In addition to the additives listed above, Daoy cells were grown aseptically in DMEM/F12 medium supplemented with 1% non-essential amino acids and 0.04 percent HEPES.

### 2.7. In Vitro Cytotoxicity Studies

The conversion of yellow MTT to purple formazan crystals by mitochondrial dehydrogenase of viable cell enzymes was used to assess the magnitude of cellular cytotoxicity exerted by CXB-loaded SLN as reported by Hussain et al. [36] and modified by Badran et al. [37]. Briefly, cancer cells were plated in 96-well plates (5 × 10^4^ cells/well) in full cell growth medium and incubated for 24 h at 37 °C under a humidified atmosphere of 5% CO_2_. The cell medium was then replaced by a cell growth medium containing 5% FBS (5% medium), buffer (control), CXB (34 µg/mL, equivalent to 89.15 µM) or equivalent from formulations 9 and 14, and was incubated for 24, 48 or 72 h at 37 °C. SLN formulations containing a CXB equivalent amounting to the above concentration were compared with both a drug-free SLN for each formulation as a negative control and a pure CXB as positive control. After incubation, medium in all test and control wells was substituted by 100 µL/well of MTT solution (0.5 mg/mL, in PBS) and incubated for further 3 h at 37 °C. Afterwards, MTT solution was exchanged with 100 µL isopropanol/well to dissolve the purple formazan crystals formed at the bottom of the wells, with shaking for at least 2 h at room temperature. Subsequently, the color intensity in the wells was measured at 549 nm with a Bio-Tek microplate reader (ELX 800; Bio-Tek Instruments, Winooski, VT, USA). To ensure the reproducibility of the results, each experiment was repeated at least three times, and the mean was calculated for comparison. The data are presented as the percentages of viable cells in the test wells compared to those of the control group. The following equation was used to calculate the cell viability [38].

% cell viability = [A549 nm of treated cells/A549 nm of control cells] × 100(7)

### 2.8. Statistical Analysis

Differences between obtained values (Mean ± SE) for the prepared formulae and the control formulae were carried out using one-way analysis of variance (ANOVA), followed by an appropriate post-hoc test in the case of the presence of a significant difference. A *p*-value less than 0.05 was considered a criterion for a statistically significant difference.

## 3. Results

### 3.1. Particle Size and Zeta Potential

**Figure 1:**
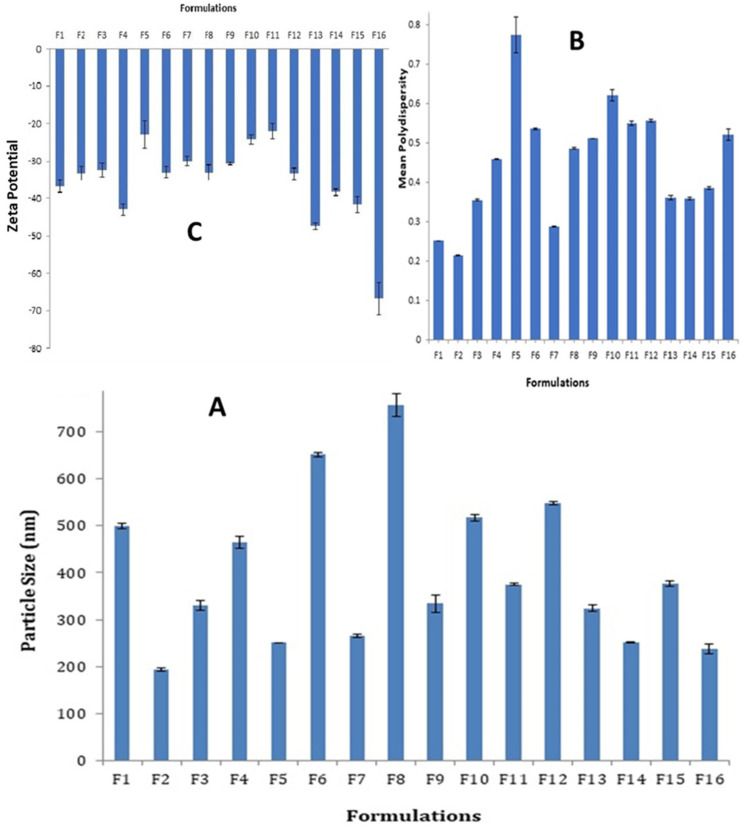
Particle sizes, size distribution, and zeta potentials of all SLN formulations: (**A**) Histogram of the mean particle sizes; (**B**) Histogram of the mean polydispersity indices; (**C**) Histogram of mean zeta potentials.

The mean particle size values of each of the prepared SLN formulations are shown in Figure 1A. The particle sizes of all SLN formulations were in the submicron range, with the highest value 757 nm ± 24.13 for F8 and the smallest value 192.6 nm ± 3.3 for F2. Increased particle size was observed when the amount of Tween 80 in SLN formulations containing Dynasan 114 (F2 and F7), stearic acid (F3 and F8), and Softisan 154 (F4 and F6) was doubled, whereas formulations containing Imwitor 308 (F1 and F5) resulted in a significantly reduced particle size (*p* < 0.001). The effect of lipid combinations on particle size appeared to be close to the estimated sizes based on those obtained with each individual lipid component in the majority of cases. As a result, the mean particle size obtained with F10 (Stearic acid + Dynasan 114) was 517.0 nm, which is very close to the arithmetic mean of 266.0 nm obtained with F7 (Dynasan 114 alone) and 757.0 nm obtained with F8 (Stearic acid alone). Similarly, the mean particle size obtained with F11 (stearic acid + Imwitor 308) was 375.0 nm, which falls between the 757.0 nm obtained with F8 (stearic acid alone) and the 250.3 nm obtained with F5 (Imwitor 308 alone). This can also be extended to F12 and F13. The type of surfactant was discovered to be a determinant factor in SLN particle sizes. Particle sizes were significantly lower in formulations made with cremophor EL (F14 and F16) compared to F13 and F15 made with Tween 80 (*p* < 0.05).

The mean polydispersity indices of all prepared SLN formulations are depicted in Figure 1B. All of the prepared SLN formulations had acceptable polydispersity indices ranging from 0.2 to 0.8, while F1, F2 and F7 had an ideal size distribution (less than 0.3).

The mean zeta potential values obtained for all prepared SLN formulations are shown in Figure 1C. The reported values ranged from (−66.7 mv ± 4.4) to (−22 mv ± 2.1). The zeta potential values were significantly reduced when the co-surfactant to surfactant ratio was reduced. Formulations F5, F6, F7 and F8 with a 1:4 ratio had significantly lower values than the corresponding formulations F1, F4, F2 and F3 with 1:2 ratios. This was also determined by comparing the zeta potential values of F13 to F9 and F15 to F11.

### 3.2. Drug Loading and Entrapment Efficiency

The entrapment efficiency and drug loading of each prepared SLN were shown in Table 2. All of the SLN formulations had high entrapment efficiency (%EE) in the range of (86.76–96.57%), with the majority exceeding 90%. The %DL of CXB in SLN was found to be within a very narrow range from 8.85% to 9.69%. Factors such as lipid type, lipid combination, surfactant type and surfactant proportion were found to be inconsistent in affecting both the %EE as well as the %DL of CXB in SLN.

### 3.3. Differential Scanning Calorimetry

**Figure 2:**
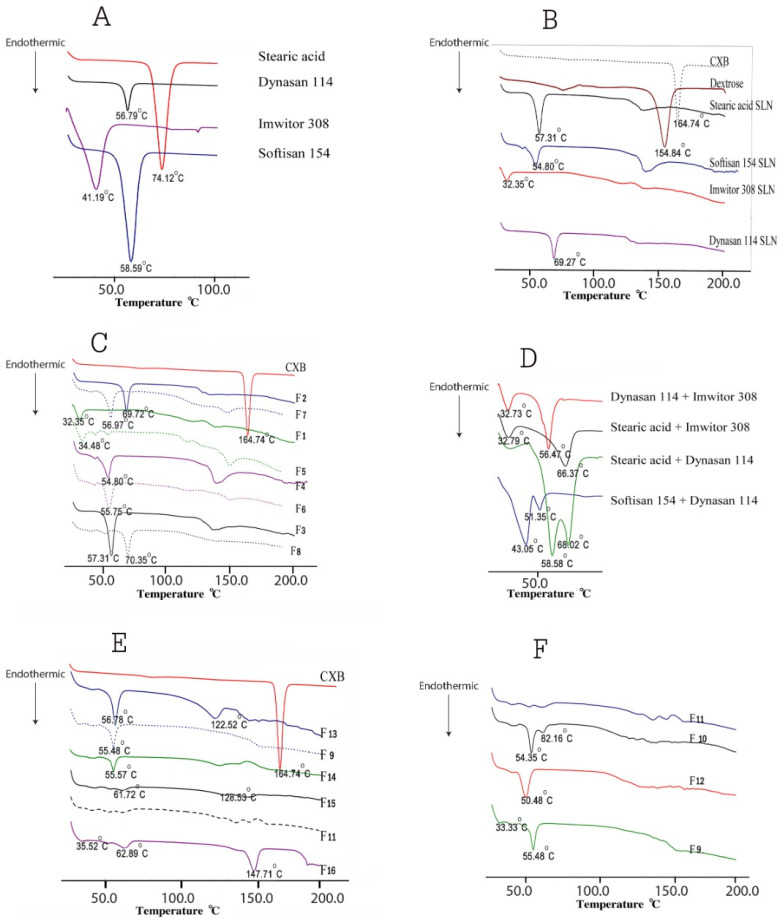
Differential scanning calorimetry for CXB, lipids, and all SLN formulations: (**A**) DSC thermograms of pure lipids: stearic acid, Dynasan 114, Imwitor 308 and Softisan 154, (**B**) DSC thermograms of pure CXB, dextrose and unloaded SLN formulations containing single lipid, (**C**) DSC thermograms of SLN formulations F1–F8, (**D**) DSC thermograms of unloaded SLN formulations containing various double-lipid combinations, (**E**) DSC thermograms of F9, F11 and F13–F16: double-lipid combination SLN formulations with various surfactant combinations, (**F**) DSC thermograms of F9–F12: double-lipid combination SLN formulations using the same surfactant combination.

Figure 2 compares DSC thermograms for all formulations to drug-free formulations, pure CXB, Imwitor 308, Dynasan 114, stearic acid and Softisan 154. Figure 2B depicts the characteristic CXB sharp melting endothermic peak at 164.64 °C, while Figure 2A depicts the melting endothermic peaks of Imwitor 308, Dynasan 114, Stearic Acid and Softisan 154 at 41.19, 56.79, 74.12 and 58.59 °C, respectively. The peak temperature and enthalpy (H j/g) for each lipid are shown in Table 3. Except for Dynasan 114, the thermograms of all SLN formulations showed a complete absence of the CXB peak, as well as a shift of the lipid endothermic peaks to lower temperatures and a reduction in their enthalpies (Figure 2C,E,F). The peak temperature was shifted to 69.27 °C, with almost no change in enthalpy. The endothermic melting peak of dextrose was observed in the majority of SLN formulations with temperature shifts to variable lower temperatures. The absence of a peak of CXB would indicate complete drug solubilization within the SLN and the presence of CXB in the amorphous state.

### 3.4. Fourier Transform Infrared Spectroscopy

**Figure 3:**
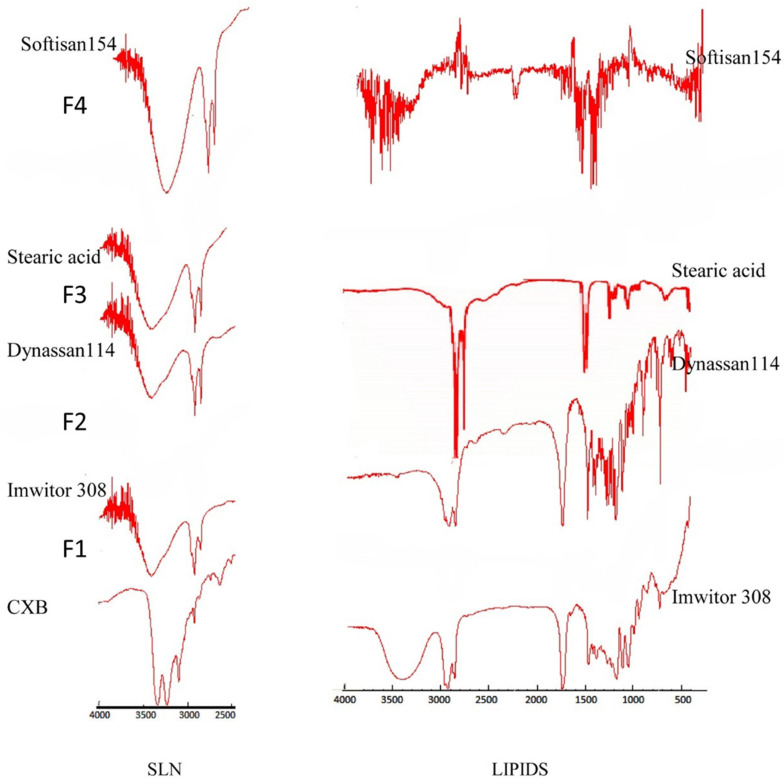
FTIR spectra of all lipids and all SLN formulations.

FTIR spectra of the pure drug, different lipids, and SLN formulations were obtained using the conventional KBr pellet method on a Nicolet 380 FTIR (Thermo electron corporation, Madison, WI, USA). Variations in spectra measurements arise as a result of an alteration in bond vibrational frequencies, which results in a frequency shift and splitting of absorption peaks. Figure 3 depicts the FTIR spectra of CXB, which shows two characteristic bands related to the NH_2_ in the NH_2_SO_2_ at 3234 cm^−1^ and 3340 cm^−1^, respectively. There was no band in this range in any of the lipids tested. The FTIR spectra of the prepared SLN formulations (F1–F4) show the disappearance of these two bands as well as the formation of a new broad band shifted toward a high wave number. It is expected that, in the presence of water and an emulsifying agent, there would be hydrogen bonding between N–H as a hydrogen donor and the carbonyl group of the glyceryl esters. This indicates a strong attachment of the drug molecules with the lipid matrix in the SLN that can enhance the drug release profile.

### 3.5. Particle Morphology 

**Figure 4:**
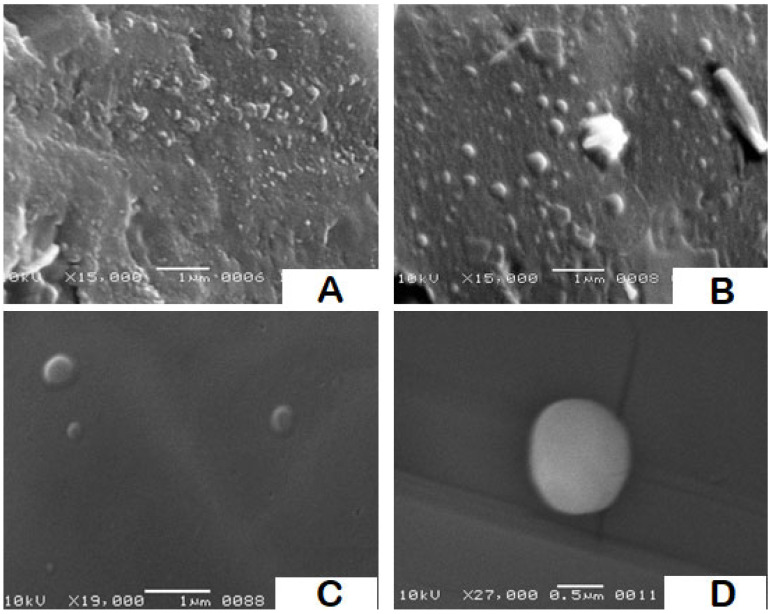
SEM photomicrographs for SLN formulations F2, F9, and F14: (**A**) a field containing a range of particles from F2 using 15,000× magnification power, (**B**) a field containing a range of particles from F9 using 15,000× magnification power, and (**C**,**D**) two high resolution fields containing few particles from F14 using magnification power 19,000× and 27,000×, respectively.

Samples from formulations F2, F9 and F14 were chosen for SEM imaging as a model to provide information on the topographic features of the particles as well as a co-indicator for particle size and size distribution. SEM images of F2 and F9 are shown in Figure 4A,B. The size dispersion of the particles is clearly visible in both images. Based on the observed fields, it is possible to conclude that the particle sizes and polydispersity indices for both formulations are comparable to the values obtained with DLS, with a slight shift to lower values, as is common. The topographical contour of the particles is demonstrated by images C and D in Figure 4, which were taken at different magnifications of a few particles from F14. The particles were semispherical in shape, with smooth to slightly rough surfaces.

### 3.6. In Vitro Release 

**Figure 5:**
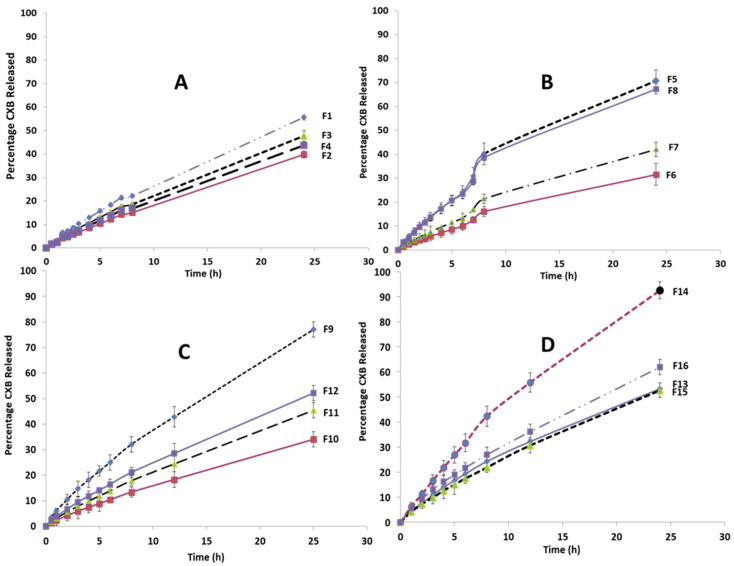
Release profile of CXB from all SLN formulation: (**A**) Effect of the type of lipid; (**B**) Effect of doubling the ratio of the surfactant (Tween 80); (**C**) Effect of using combination of lipids; (**D**) Effect of using Cremophor EL as a surfactant. NB: All of the lines were drawn by connecting all graph points to form the observed release profile shape, rather than by fitting to the data points.

The in vitro release of CXB from all SLN formulations was studied for 24 h using the dialysis bag method in a phosphate buffer pH 6.8 medium. Pure CXB showed a rapid release with a magnitude of approximately 93% in the first three hours. This demonstrates the utility of using a dialysis bag to monitor the release profile of CXB from SLN formulations. Figure 5 shows the CXB release profiles from the various SLN formulations. During the first 6 h, no burst drug release was observed for any of the SLN formulations.

Figure 5A compares the CXB release profiles of different SLN formulations (F1, F2, F3, F4) containing a single lipid component. F1 (Imwitor 308) had the highest cumulative CXB% released after 24 h, followed by F3 (stearic acid), F4 (Softisan 154), and the lowest F2 (Dynasan 114). As shown in Figure 5B, increasing the Tween 80 concentration to 20% of the total lipids resulted in a significant increase in the cumulative CXB% released for the four tested lipids to varying degrees. This increase was nearly 70% after 24 h for formulations containing Imwitor 308 (F5) and stearic acid (F8). The release profiles of four formulations (F9–F12) containing multiple binary lipid admixtures and formulated with 20% Tween 80 are shown in Figure 5C. F9, which contained Dynasan 114 and Imwitor308, had the highest release rate, with a cumulative CXB release rate of 77% after 24 h; thus, it is considered the most favorable profile for per-oral colonic delivery. F10 (Dynasan 114 and stearic acid), F11 (Imwitor 308 and stearic acid) and F12 (Softisan 154 and Dynasan 114) all had significantly lower release rates than F9, with 24 h cumulative CXB% release rates of 34%, 45% and 52%, respectively (*p* < 0.05).

Figure 5D compares the CXB release profiles of four formulations (F13–F16) that differ in surfactant type and percentage, using either 20% Cremophor EL or 10% Tween 80 in SLN formulations containing either an Imwitor 308 and Dynasan 114 combination (F13 and F14) or an Imwitor 308 and a stearic acid lipid combination (F15 and F16). After 24 h, F14 had the fastest release rate, with an extent of 92.6%, followed by F16 (62%). When the CXB release profiles of F14 were compared to those of F9 and F13, which had the same lipid combination, it was discovered that 20% Cremophor EL significantly increased both the rate and extent of CXB release. This was also confirmed by the higher release rate obtained with F16 compared to F15 and F11, despite the fact that the three formulations have the same lipid composition. The degree of enhancement in the drug release rate obtained with Cremophor EL appeared to be clearly dependent on the type of lipid components, as it was significantly greater with the Dynasan 114 and Imwitor 308 combination than the Imwitor 308 and stearic acid combination. It is also clear that lowering the percentage of Tween 80 resulted in a significant decrease in the rate of CXB release. This can be concluded by comparing the release profiles of F13 and F9, as well as F15 and F11.

CXB release kinetics were investigated by fitting the fraction drug released from the prepared SLN against time data to the MULTI computer program using the Higuchi and Korsemeyer–Peppas models, with AIC and SSR used to determine which model fit best. Korsemeyer–Peppas models with n values greater than 0.5 fit better, according to the results. This finding implies that CXB release did not follow the Higuchi or Fikian diffusion pathways. According to the Korsemeyer–Peppas model, the drug release follows non-Fikian release kinetics with an n in the range of 0.45 to 0.89.

### 3.7. In Vitro Cytotoxicity 

**Figure 6:**
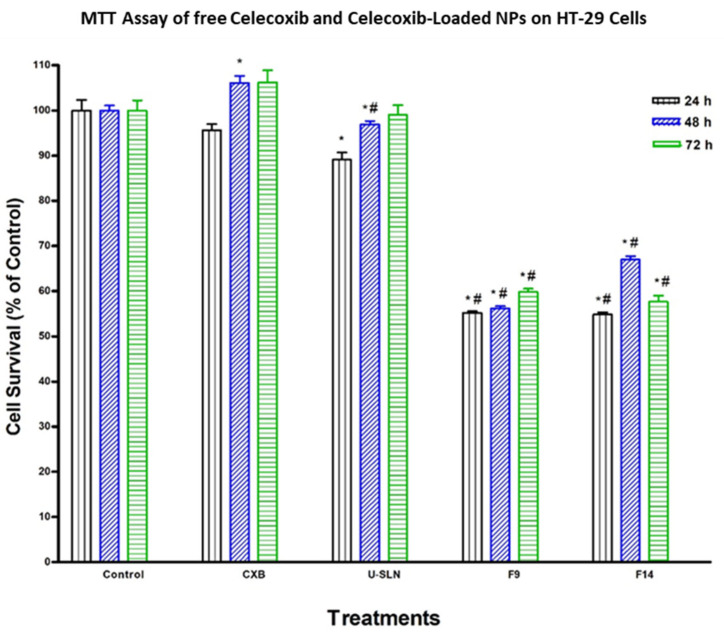
Effect of CXB and CXP–SLN on colorectal cancer cells. HT-29 cells were treated with indicated formulas of CXB–SLN (F9, F14), unloaded SLN (U-SLN), pure CXB or buffer (control) for 24, 48 or 72 h. Cell viability was determined by MTT assay, as indicated in Section 2. At the end of the assay, the absorbance at 549 nm was read on a microplate reader. Significant differences between treatments and control and CXB were analyzed by ANOVA followed by *t*-test. * *p* < 0.05 compared with control (0 µM). # *p* < 0.05 compared with CXB.

**Figure 7:**
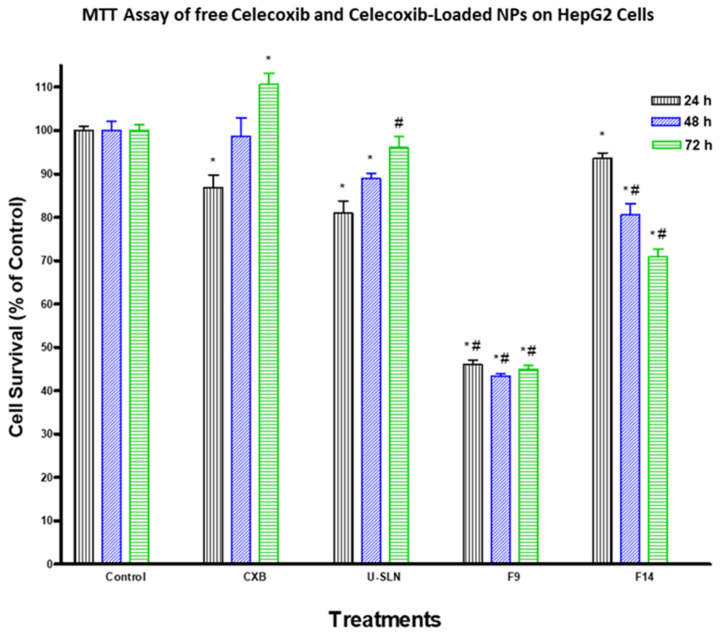
Effect of CXB and CXP–SLN on hepatocellular carcinoma cells. HepG2 cells were treated with indicated formulas of CXB–SLN (F9, F14), unloaded SLN, and pure CXB or buffer (control) for 24, 48 or 72 h. Cell viability was determined by MTT assay, as indicated in Section 2. At the end of the assay, an absorbance of 549 nm was read on a microplate reader. Significant differences between treatments and control and CXB were analyzed by ANOVA followed by *t*-test. * *p* < 0.05 compared with control (0 µM). # *p* < 0.05 compared with CXB.

**Figure 8:**
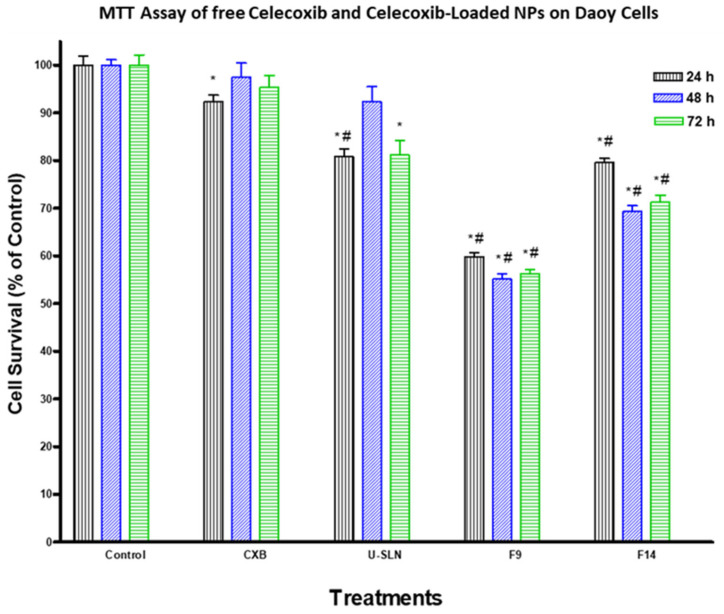
Effect of CXB and CXP–SLN on medulloblastoma cells. Daoy cells were treated with indicated formulas of CXB–SLN (F9, F14), unloaded SLN (U-SLN), pure CXB or buffer (control) for 24, 48 or 72 h. Cell viability was determined by MTT assay, as indicated in Section 2. At the end of the assay, an absorbance of 549 nm was read on a microplate reader. Significant differences between treatments and control and CXB were analyzed by ANOVA followed by *t*-test. * *p* < 0.05 compared with control (0 µM). # *p* < 0.05 compared with CXB.

The effect of loading CXB in SLN (CXB–SLN) on its cytotoxicity was investigated by using the MTT assay to assess its anti-proliferative effects, as described in Section 2. After 24, 48 and 72 h of incubation, the cytotoxic effects of pure CXB and CXB-SLN formulations F9 and F14 in three cancer cell lines, HT-29, HepG2 and Daoy, were investigated. Figure 6, Figure 7 and Figure 8 show that pure CXB has no cytotoxicity against the CRC HT-29 cell line at any time point. After incubating HT-29 cells with CXB for 24, 48 and 72 h, there was only 4.4%, zero percent growth inhibition and zero percent inhibition, respectively. Nonetheless, CXB-SLN formulations containing Dynasan 114 and Imwitor 308, F9 (20% Tween 80), and F14 (20% Cremophor EL) demonstrated significantly higher cytotoxicity against HT-29 cells than pure CXB and the buffer control (*p* < 0.0001). However, after only 24 h of incubation, the effect was observed in the three F9 cell lines. Figure 6 depicts that the longer incubations (48 and 72 h) had no effect on the inhibition of HT-29 growth. This suggests that the CXB formulas had the greatest effect on HT-29 after 24 h. F9 also inhibited the proliferation of HepG2 and Daoy cells, whereas pure CXB had only modest activity against these cells after 24 h, with no significant increase in effect after 48 or 72 h (Figure 7). This corresponds to the in vitro release profiles of the two formulations, which showed that the majority of the loaded CXB was released within 24 h. The most sensitive cell line to F9-induced cell death was HepG2, followed by HT-29 and Daoy cells. Only after 48 and 72 h did the formula F14 significantly improve CXB activities against HepG2 cells, whereas significant cytotoxicity was observed against Daoy cells in all three incubation periods. Surprisingly, the effects of F14 on the Daoy cell line were time-dependent, with activity increasing over time (Figure 8). HT-29 was the most sensitive cell line to F14-induced cell death, while HepG2 was the least sensitive.

## 4. Discussion

The incorporation of CXB into SLN formulations was optimized using a variety of lipids and surfactants to achieve optimal formulations with superior properties such as high EE, low nanosize, and a uniform release profile suitable for colon delivery. The inverse correlation between the melting point of the lipid component and the particle size of SLN that was pointed out by Muhlen et al. [39] was only observed in our results when a low surfactant ratio was used (10%). In the case of a high surfactant ratio (20%), no correlation was observed, because F5, containing the lowest melting point lipid (Imwitor 308), had the smallest particle size, and F8, which formulated with the highest melting point lipid (stearic acid), had the largest particle size.

Our findings revealed a link between increasing the surfactant ratio and the increase in the particle size of the formulated SLN. This is consistent with the results of Zhao et al. [40], who found that increasing the surfactant/co-surfactant ratio resulted in an increase in particle sizes due to the expansion of the interfacial film. According to Asasutajarit et al. [41], excess surfactant accumulation on the SLN surface resulted in an increase in SLN particle sizes. Vivek et al. [42], on the other hand, discovered that increasing surfactant concentration by more than 1.5% has no effect on particle size. The significantly lower particle sizes obtained with Cremophor El formulations compared to Tween 80 formulations is in agreement with the results obtained by Patil et al. [43]. This can be attributed to the larger hydrophobic part of Cremophor EL, as indicated by the lower HLB value 13.5 versus 15 for Tween 80. 

The polydispersity index is a ratio that gives a descriptive measure of the homogeneity of the particle size distribution in each system. Pathak et al. reported that polydispersity values less than 0.3 are considered ideal, whereas polydispersity values less than 0.1 are considered a monodisperse [34]. The polydispersity indices lay within the average range commonly reported for SLN prepared with ultrasonication [44]. SEM images were used to evaluate the particle morphology for three formulations. F2, the formula with the smallest particle size, as well as F9 and F14, demonstrated the best attributes and were chosen for cytotoxicity testing. Overall, the sizes and polydispersity obtained by DLS concurred with those obtained by SEM.

The obtained high zeta potential values for the majority of the prepared SLN formulations give rise to the high physical stability of the prepared SLN by this method and their ability to resist agglomeration upon storage. It is commonly agreeable that values above ±30 mv are required for full electrostatic stabilization [45]. The incorporation of sodium deoxycholate as a co-surfactant was intended for this purpose. Fillery-Travis et al. [46] recommended the use of an anionic surfactant such as sodium deoxycholate to enhance particle stability and produce high zeta potential values. It has been demonstrated that the %EE and %DL in the SLN formulation can be significantly affected by the type of lipid and/or surfactant [47,48]. The obtained high %EE and %DL results with our CXB–SLN formulations can be attributed to the high lipophilic properties of CXB resulting in high solubility in lipids and the use of an adequate surfactant ratio.

The absence of melting the endotherm of CXB in the DSC thermograms of all SLN formulations is an indication of the solubilization of CXB inside the lipid. This is in agreement with many reports in the literature [33,49,50]. 

The broadening in the peak shape observed in many of formulated thermograms is common due to the variation in particle size in the tested sample as well as the existence of multiple lipid polymorphs [51,52]. Bunjes et al. [53] found that the incorporation of a combination of lipid glycerides has been associated with an alteration in crystallization and melting behavior as well as a polymorphic transition. On the other hand, complex lipid mixtures are not capable of forming particles with a defined progression in their thickness, thus a diffused lattice is produced. In this case, a broad melting incident with a comparatively low transition temperature appeared [54]. The observed decrease in the melting enthalpies of lipids in SLN was also reported for colloidal solid triglyceride [55]. Other reports indicated that the decrease in the melting enthalpies can be caused by the low drug loading [56,57,58].

The absence of the CXB characteristic bands for both NH_2_ and NH_2_SO_2_ from the FTIR spectrum for all SLN formulations indicated the high conjugation of the CXB with the lipid matrix by the formation of hydrogen bonding between the carbonyl of the fatty acid ester with the amine group in CXB. The formation of hydrogen bonds resulted in the disappearance of the N–H characteristic bands, with the formation of a stretching, broad band shifting towards a higher frequency [59]. 

Because it is commonly agreed upon in the design of perorally-administered colon drug delivery to incorporate an enteric system to eliminate the possibility of high variability in gastric emptying, which may impart significant pre-colonic drug release, the drug release studies were conducted in a phosphate buffer pH 6.8 medium. Our SLN formulation is intended to be incorporated into an enteric coated capsule system, ensuring release in the middle and terminal regions of the intestine while minimizing interactions with food and digesting enzymes. In one of our previous studies, a typical example of such an enteric capsule was presented, and it demonstrated high in vivo efficiency [60]. Generally, the drug release profile from all SLN formulations have demonstrated a number of beneficial attributes such as a low burst effect and a uniform rate, which comprises a common limitation for SLN. Such a biphasic mode of release has been identified in many reports involving the release of various drugs including prednisolone, etomidate, and tetracaine from different SLN formulations [56,61,62,63]. The Muhlen biphasic model explained the burst drug release by drug molecules expelled on the surface of SLN crystals, as well as the slow release phase to the embedded drug inside the SLN core [56]. The melt emulsification method used to prepare CXB–SLN has successfully produced SLN with a low burst release profile and homogenous distribution of CXB within the various layers of the SLN matrix, which is in agreement with the observed high %EE. This also correlates well with the formation of hydrogen bonding between CXB and the lipid component as postulated from the absence of the characteristic NH_2_ band from the CXB-SLN FTIR spectra. The slower rate of CXB release from F2-containing Dynasan 114 compared with the other mono-lipid SLN formulations has been observed in other work and can be attributed to its triglyceride composition with a single fatty acid (myristic acid) which has been shown to allow for a deep intake of drugs within the layers of the SLN and the delay in transformation of the unstable α form to the stable β form [64]

Increasing the surfactant concentration has a clear impact on CXB’s drug release profile. It has been proposed that increasing the surfactant concentration can improve the drug release profile by increasing the drug’s solubility in the water phase [47]. This is consistent with the results obtained with F5, F7, and F8. 

The observed higher cumulative % release with F9, containing a combination of Dynasan 114 and Imwitor 308, is in agreement with the results reported by Alarifi et al. [65] for the release of ciprofloxacin from SLN composed of stearic acid/Imwitor 900 or Dynasan 118 combinations. The significant increase in the cumulative CXB% release when using Cremophor EL as a surfactant is in agreement with Kiss et al. [66], who concluded that Cremophor EL causes a reduction in the melting point of lipid mixture, resulting in a reduction in their crystallinity index.

The kinetic analysis of the drug release pattern shows a high compliance with the non-Fickian model that was confirmed by the values of Korsemeyer–Peppas’ exponent “n”. There was no burst release, indicating that the drug had not been adsorbed on the particles’ surfaces. In the formulation, the drug was divided between the lipid core and the micellar coat. The drug on the micellar coat was released first, and the solid lipid softened and slowly released the drug, according to the release profile. Silva et al. [67] and Kushwaha et al. [68] reported similar non-Fickian release kinetics for risperidone and isoniazid loaded SLN, respectively. On the other hand, the release of olanzapine from SLN composed of three lipids, Precirol ATO 5, glyceryl tristearate and Witepsol E85, was observed to follow Higuchi’s release kinetic model with a burst effect [42]. The same conclusion was reached regarding the release of etodolac from tristearin SLN [69].

Consistent with the study aim, both F9 and F14 were chosen for the in vitro cytotoxicity study. They had the highest cumulative CXB% released of any formulation, with the least amount released during the first 4 h, implying the least pre-colonic drug release based on the average GI transit time. As a result, when compared to other SLN formulations, these two formulations have the highest colon targeting affinity for CXB. Furthermore, both have good physical properties in terms of particle size, dispersity, charge and %EE. The cytotoxic activity of CXB-loaded SLN and pure CXB was observed to be time-independent. Other studies do not support the very low activity of pure CXB on HT29 and DOAY cells. Wang et al. [70], for example, reported that a similar CXB concentration inhibited the growth of HT-29 by 14, 23 and 23% after incubation for 24, 48 and 72 h, respectively. This could be due to the fact that we used a higher number of HT-29 cells (5 × 10^4^ vs. 5 × 10^3^). It is worth noting that the HT-29 cell death obtained from F9 (45%) is significantly higher than the 23 and 25% cell death reported for CBX encapsulated in large unilamellar vesicles (LUVs) and EGFR-targeted immune-liposomes, respectively [71]. F9-induced cell death is also significantly higher than that observed with CXB-loaded PEGylated liposomes by Erdoğ et al. [72], who demonstrated that CXB-loaded PEGylated liposomes reduced the viability of HT-29 cells by about 0, 35 and 45% after 24, 48 and 72 h of incubation using a final liposomal CXB concentration of 100 µM (higher than our concentration of 89.15 µM). Despite the fact that CXB has been reported by many researchers to inhibit the growth of HepG2 [73] and Daoy cells [74], we believe we are the first to show the effects of CXB nanoparticles on these two cell lines. The significantly higher cytotoxicity observed with F9 than with F14 in the three cell lines suggests that Tween 80-stabilized SLN increases CXB cytotoxicity more than Cremophor EL-stabilized SLN, however the mechanism is unknown. This could be because Tween 80 has a greater ability than Cremophor EL to cause adhesive effects with cell membranes. Despite the high toxicity of Cremophor EL, it was used in relatively low concentrations for SLN stabilization. The induced increase in CXB release was not associated with increased cytotoxicity. F9, a new CXB-SLN formulation, demonstrated significant cytotoxicity in three resistant cancer cell lines, as well as a high colon targeting ability via per-oral administration and unique physical properties of high stability and entrapment efficiency. Combine this with the well-known nanomedical benefits in solid tumor cancers, such as increased tumor diffusivity and passive targeting ability due to enhanced permeation and retention phenomena [23,75]. Other tissues’ exposure to CXB, on the other hand, would be greatly reduced, as would all of the associated adverse effects. Increased lymphatic uptake of SLN via the payer’s patches in the distal part of the GIT has the important benefit of providing greater resistance to cancer metastasis, which occurs primarily through the lymphatic system [76]. By incorporating methotrexate into SLN, lymphatic uptake has been used to improve methotrexate bioavailability [77]. As a result, our proposed system is a very promising option for colon cancer prevention and treatment, either alone or in combination with other approaches such as mitochondrial targeting, osmotic pressure, and immunotherapy [75]. 

F9 is regarded as the best SLN formulation because it combines all beneficial properties such as highly significant cytotoxic effects in three cancer cell lines, robust physical properties, uniform drug release, and the absence of components with potential adverse effects and toxicity. More research is needed to assess the in vivo performance and stability of our proposed SLN system in order to articulate the observed anticancer activity and potential mechanisms, as well as to determine the full drug safety profile.

## 5. Conclusions

The presented melt emulsification method was successful in optimizing CXB loaded SLN with superior physical properties such as uniform low particle sizes, high surface charge indicating high stability, and high CXB entrapment and loading capacity. They had consistent drug release profiles with no bursts and uniform rates that lasted 24 h. Although both the F9 and F14 release profiles can be used for specific colon delivery of CXB after per-oral administration without the need for any additional complicated formulation steps, F9 is considered the optimum formulation because it combines the highest in vitro anticancer activity with biocompatible components. Our study introduces a novel orally administered CXB-loaded SLN that overcomes the common limitation of high burst drug release and can specifically target the drug to the colon. The prepared SLN successfully increased CXB activity against a resistant COX-2-bearing colon cancer cell line as well as two other resistant cancer cell lines. This enables the use of higher doses of CXB without the fear of the COX-2 inhibitor’s adverse effects. Such a system can be translated into an effective therapeutic option for both colon cancer prevention and treatment.

## Figures and Tables

**Table 1:** Compositions of the prepared CXB-Loaded SLN.

Formula	Lipid				Surfactant/Co-Surfactant		
	Stearic Acid	Dynasan 114	Imwitor 308	Softisan 154	Tween 80	Cremophor EL	Sodium Deoxycholate
F1	--	--	450 mg	--	45 mg	--	22.5 mg
F2	--	450 mg	--	--	45 mg	--	22.5 mg
F3	450 mg	--	--	--	45 mg	--	22.5 mg
F4	--	--	--	450 mg	45 mg	--	22.5 mg
F5	--	--	450 mg	--	90 mg	--	22.5 mg
F6	--	--	--	450 mg	90 mg	--	22.5 mg
F7	--	450 mg	--	--	90 mg	--	22.5 mg
F8	450 mg	--	--	--	90 mg	--	22.5 mg
F9	--	225 mg	225 mg	--	90 mg	--	22.5 mg
F10	225 mg	225 mg	--	--	90 mg	--	22.5 mg
F11	225 mg	--	225 mg	--	90 mg	--	22.5 mg
F12	--	225 mg	--	225 mg	90 mg	--	22.5 mg
F13	--	225 mg	225 mg	--	45 mg	--	22.5 mg
F14	--	225 mg	225 mg	--	--	90 mg	22.5 mg
F15	225 mg	--	225 mg	--	45 mg	--	22.5 mg
F16	225 mg	--	225 mg	--	--	90 mg	22.5 mg

**Table 2:** Percentage drug entrapment efficiency (%EE) and drug loading (%DL) of all prepared SLN formulations.

Formulation	%EE	%DL
F1	90.9 ± 1.12	9.17 ± 0.12
F2	90.5 ± 0.62	9.14 ± 0.06
F3	95.0 ± 0.72	9.55 ± 0.07
F4	93.7 ± 1.63	9.42 ± 0.62
F5	88.7 ± 3.10	8.97 ± 0.32
F6	93.0 ± 0.93	9.36 ± 0.39
F7	93.3 ± 2.09	9.38 ± 0.21
F8	92.0 ± 4.2	9.27 ± 0.16
F9	86.8 ± 1.59	8.97 ± 0.16
F10	87.4 ± 0.98	8.85 ± 0.10
F11	92.2 ± 1.82	9.29 ± 0.18
F12	94.1 ± 2.67	9.46 ± 0.28
F13	96.6 ± 1.19	9.96 ± 0.12
F14	95.5 ± 2.29	9.59 ± 0.23
F15	96.1 ± 3.38	9.65 ± 0.35
F16	95.4 ± 2.04	9.58 ± 0.21

**Table 3:** DSC peak temperatures and enthalpies (Δ H j/g) for some pure lipids and their analogues inside SLN formulations.

Lipid	Composition	Peak Temp °C	Δ H j/g	SLN Peak Temp °C	SLN Δ H j/g
Imwitor 308	Mono-glyceryl ester with caprylic acid, 8-C fatty acid	41.19	−234.93	32.65	−27.58
Dynasan 114	Tri-glyceryl ester with myristic acid, 14-C fatty acid	56.79	−44.07	69.27	−45.26
Softisan 154	Tri-glyceryl ester of a blend of 16-C and 18-C saturated fatty acid	58.59	−234.96	54.8	−40.97
Stearic acid	Fatty acid containing 18-C saturated hydrocarbon chain	74.12	−238.03	57.31	−93.28
